# Autism Spectrum Disorder: What Do We Know and Where Do We Go?

**DOI:** 10.3390/brainsci15091010

**Published:** 2025-09-18

**Authors:** Gerry Leisman, Robert Melillo

**Affiliations:** 1 Movement and Cognition Laboratory, Department of Physical Therapy, University of Haifa, Haifa 3498838, Israel; drm@drrobertmelillo.com; 2 Resonance Therapeutics Laboratory, Department of Neurology, Universidad de Ciencias Médicas de la Habana, Havana 11600, Cuba; 3 Center for Developing Minds, Rockville Centre, NY 11570, USA

**Keywords:** autism spectrum disorder (ASD), neurodevelopment, brain connectivity, genetic and metabolic mechanisms, immunological dysregulation, developmental trajectories

## Abstract

Autism spectrum disorder (ASD) is a complex neurodevelopmental condition that manifests in early childhood and persists throughout an individual’s life. Characterized by a range of symptoms affecting social interaction, communication, and behavior, ASD presents a spectrum of varying degrees of severity and presentation. Recent research emphasizes the importance of understanding the diverse manifestations of ASD across different populations. Core features include social communication differences and restricted and repetitive behaviors (RRBs), often linked to co-occurring conditions such as anxiety and ADHD. The study of ASD has evolved significantly, highlighting the need for individualized approaches to diagnosis and intervention. This paper explores current knowledge on ASD, examining the latest research findings and discussing future directions for improving the lives of those affected by the disorder. The purpose is to present a map of the field and an evidence-strength framing of what is known and unknown, and where the evidence is equivocal. Key areas of focus include behavioral, psychological, genetic, metabolic, immunological, and neurological features, as well as developmental and maturational factors. The aim is to provide a comprehensive overview of what is known, what remains unclear, and where future research should be directed.

## 1. Introduction

Autism spectrum disorder (ASD) is a heterogeneous neurodevelopmental condition that emerges in early childhood and persists across the lifespan. It is characterized by two core domains: persistent social communication differences and restricted, repetitive patterns of behavior, interests, or activities, with onset in early development and clinically significant functional impact. Unlike many other neurodevelopmental conditions, ASD is defined less by uniformity than by marked diversity. Its manifestations range from individuals who require substantial daily support to those who function independently but continue to experience challenges in social reciprocity, communication, and adaptive behavior. This variability has led to the recognition of ASD as a spectrum rather than a single entity.

The complexity of ASD lies not only in its behavioral presentation but also in its developmental, psychological, genetic, metabolic, immunological, and neurological dimensions. Research consistently demonstrates that social communication differences include reduced reciprocity, atypical nonverbal signaling, and challenges in peer relationships, though these are often better conceptualized as qualitative differences rather than uniform deficits. Restricted and repetitive behaviors (RRBs) further illustrate the heterogeneity of ASD, spanning from stereotyped movements and insistence on sameness to sensory reactivity differences and circumscribed interests. Importantly, these domains interact with co-occurring conditions such as anxiety, attention-deficit/hyperactivity disorder (ADHD), and sleep problems, which shape adaptive outcomes and quality of life.

Over the past two decades, advances in longitudinal cohort studies, infant sibling prospective designs, and large-scale meta-analyses have substantially altered the field’s understanding of ASD trajectories. Early markers can now be identified within the first year of life, long before clinical diagnosis, and developmental outcomes vary widely, with some individuals showing improvement with supports and others maintaining stable differences despite gains in cognitive ability. The recognition of camouflaging strategies, particularly among females and gender-diverse individuals, has further highlighted the need to reconsider how ASD is detected, conceptualized, and supported. While camouflaging may allow some individuals to navigate social contexts more effectively, it is also associated with significant costs, including increased anxiety, depression, and autistic burnout.

In parallel, progress in genetics, neuroimaging, immunology, and systems neuroscience has reframed ASD as a multisystem condition. Genome-wide association studies and exome sequencing implicate both common polygenic variation and rare de novo mutations in pathways central to synaptic scaffolding, transcriptional regulation, and chromatin remodeling. Neuroimaging studies have shifted the field from a focus on isolated brain regions to an emphasis on connectivity and network dynamics, with graph theory analyses revealing atypical patterns of integration and segregation that may underlie cognitive and behavioral profiles. Moreover, evidence of mitochondrial dysfunction, oxidative stress, and immune dysregulation suggests that metabolic and immunological processes intersect with neurodevelopmental pathways in ASD.

This growing body of evidence underscores that ASD cannot be understood solely as a set of behavioral differences but must be conceptualized as a condition with diverse biological underpinnings, developmental trajectories, and contextual influences. Despite this progress, substantial uncertainties remain: the causal significance of metabolic and immune abnormalities, the long-term developmental impact of camouflaging, and the extent to which neurobiological markers can be translated into clinically actionable interventions. The aim of this review is therefore not to provide an exhaustive account of every finding in the literature, but rather to integrate across behavioral, psychological, genetic, metabolic, immunological, neurological, and developmental domains. Specifically, we seek to delineate what is well established, what remains equivocal, and what is unknown. In doing so, we highlight both the scientific progress that has reshaped the field and the persistent gaps that must be addressed to improve the lives of autistic individuals and their families.

## 2. Behavioral Features

### 2.1. Core Diagnostic Domains and Contemporary Refinements

ASD is defined by two behavioral domains: persistent social communication differences and restricted, repetitive patterns of behavior, interests, or activities (RRBs), with onset in early development and clinically significant functional impact [1]. Recent work emphasizes the heterogeneity of these domains across development, sex, language level, and cognitive profile [2,3]. Social communication differences include reduced reciprocity, atypical nonverbal signaling, and differences in peer relationships, but may also represent qualitatively atypical reciprocity rather than a “deficit” [4]. RRBs span stereotypies, insistence on sameness, sensory reactivity differences, and circumscribed interests; factor-analytic work supports separable clusters with distinct links to anxiety, ADHD traits, and adaptive outcomes [5,6].

### 2.2. Developmental Timing and Early Behavioral Markers

Prospective infant sibling studies identify prodromal social-attention differences in the first year that precede diagnosis at 24–36 months [7]. By toddlerhood, language profiles range from minimal speech to advanced syntax with pragmatic differences [8]. Sensory reactivity differences are evident by 12–18 months and predict later anxiety and RRB intensity [9]. Longitudinal data show divergent growth curves—some children improve with supports, while others maintain stable differences despite IQ gains [10].

### 2.3. The Autistic Presentation Across the Lifespan

In childhood, key features include literal interpretation, circumscribed interests, difficulty with group transitions, and sensory-related distress. Executive function differences manifest as challenges with multi-step directions and flexibility [11].

In adolescence, increasing social complexity coincides with compensatory camouflaging, which is linked to anxiety, depression, and burnout [12,13]. Identity formation and deep focus on interests (monotropism) can also emerge [14].

In adulthood, core traits persist but recontextualize; routines can be stabilizing for employment, but sensory environments may be disabling [15]. Autistic burnout—chronic exhaustion and functional loss following prolonged masking—has been recognized as clinically significant [16].

Older adulthood remains understudied, though preliminary reports highlight the persistence of autistic traits with increased medical comorbidity [17].

### 2.4. Sex, Gender, and Camouflaging

Sex-linked presentation differences contribute to diagnostic delays in girls, women, and gender-diverse individuals [3,18]. Girls often exhibit fewer overt RRBs and more socially conforming interests, alongside higher camouflaging [12,13]. Camouflaging carries costs—greater internalizing symptoms, self-injury risk, and later diagnosis despite equivalent traits. Assessments that probe sensory differences and insistence on sameness are critical.

### 2.5. Co-Occurring Conditions and Behavior

Behavior is shaped by co-occurring conditions. Meta-analyses report high prevalence of ADHD (≈30–60%), anxiety (≈20–40%), depression (≈10–20%), and sleep problems (>50%) [19,20,21]. Adaptive behavior is often below expectations for IQ, with increasing divergence across development [22]. Irritability and self-injury correlate with sensory hyperreactivity, communication mismatch, sleep disturbance, and anxiety [23]. Gastrointestinal and pain-related conditions often manifest behaviorally, making systematic medical screening vital [24].

### 2.6. Sensory Processing and Motor Behavior

Sensory processing differences are now considered core. Hyper-/hyporesponsivity and seeking behaviors predict outcomes and show distinct neural correlates [25]. Motor differences (dyspraxia, atypical gait, fine-motor deficits) are common and relate to social communication via shared coordination mechanisms [26].

### 2.7. Language, Communication, and Pragmatics

Recent emphasis is on pragmatic language: inferencing, figurative language, and topic management [27]. Individuals may have strong structural language but atypical prosody or information-dense monologues. AAC use across ability levels is rising and improves participation [28].

### 2.8. Behavior in Context: The Double-Empathy Framework

Behavior reflects a bidirectional mismatch between autistic and non-autistic communication. Evidence shows autistic–autistic interactions foster greater rapport, while misunderstandings are mutual in mixed dyads [29,30]. This supports interventions targeting environments rather than exclusively individuals.

### 2.9. Behavioral Heterogeneity and Data-Driven Subtyping

Machine learning approaches reveal stable behavioral clusters (e.g., high-RRB/sensory; high-anxiety/camouflaging) that predict support needs and may map onto biological pathways [6,31]. These approaches move beyond severity-based “levels.”

### 2.10. Measurement Considerations

While the Autism Diagnostic Observation Schedule, Second Edition (ADOS-2) and Autism Diagnostic Interview-Revised (ADI-R) remain standards, research highlights context sensitivity, cultural bias, and the need for multi-informant assessment [32]. New approaches, including ecological momentary assessment, wearables, and eye-tracking, offer finer resolution of fluctuating states [33].

### 2.11. What Is Clear vs. Unclear

**Clear:** heterogeneity of RRBs, sex/gender effects and camouflaging, persistence of traits across the lifespan, autistic burnout, and double-empathy effects.

**Unclear:** biological mapping of subtypes, long-term consequences of camouflaging and burnout, robust ecological metrics, and behavioral features in older adulthood.

## 3. Psychological Features

### 3.1. Cognitive Profiles and Variability

ASD is not associated with a single cognitive profile, but rather a spectrum of abilities and styles. While a minority of individuals present with intellectual disability (ID), others demonstrate average or above-average IQ, with distinct strengths in areas such as systemizing, detail focus, or pattern recognition [2,34]. These strengths coexist with differences in executive functioning, theory of mind (ToM), and central coherence, though these constructs are now viewed as probabilistic rather than universal deficits [4]. Meta-analytic findings suggest that executive dysfunction, particularly in flexibility and planning, is common but variable [11].

### 3.2. Theory of Mind and Social Cognition

Classic accounts highlight ToM differences as central to ASD, but contemporary research underscores heterogeneity and the contextual nature of ToM performance. Many autistic individuals can succeed on explicit ToM tasks but may differ in spontaneous or real-time social cognition [35]. Difficulties may be driven not by inability but by differences in social motivation, attention allocation, or double-empathy effects [36,37]. Social cognition is further shaped by co-occurring anxiety and language level, complicating interpretation [38].

### 3.3. Executive Functioning

Executive function (EF) differences are among the most replicated psychological features of ASD. Meta-analyses show significant group-level differences in flexibility, working memory, and inhibition, but preserved or enhanced performance in attention to detail and rule-based reasoning [11,39]. Developmental studies show EF differences persist into adolescence and adulthood, predicting adaptive functioning and quality of life [40]. EF training interventions show modest benefits but require ecological adaptation for daily-life relevance [41].

### 3.4. Emotional Processing and Regulation

Autistic individuals often show heightened emotional reactivity and differences in recognition of facial affect, particularly subtle or ambiguous expressions [42]. However, evidence indicates that context, sensory load, and interoceptive accuracy mediate these effects [43]. Emotion regulation difficulties, including reliance on less adaptive strategies (suppression, avoidance), are linked to internalizing conditions such as anxiety and depression [44]. Alexithymia, often comorbid with ASD, accounts for a significant portion of observed emotion recognition differences [45].

### 3.5. Attention and Perceptual Processing

ASD is characterized by atypical attentional allocation. Eye-tracking studies consistently reveal reduced fixation on faces but enhanced attention to objects, geometry, or detail-rich scenes [46,47]. Perceptual processing favors local detail over global integration (weak central coherence), but this bias confers advantages in visual search, pattern detection, and certain STEM skills [48]. Neural data suggest enhanced low-level perceptual processing and reduced top-down modulation, supporting a predictive coding framework for perception in ASD [49].

### 3.6. Cognitive Styles and Strengths

Cognitive diversity is increasingly emphasized. Monotropism theory suggests deep, sustained focus on restricted domains, explaining both restricted interests and exceptional skills [50]. Systemizing theory highlights strengths in rule-based reasoning and pattern detection, while empathizing-systemizing imbalances explain sex differences and overlap with STEM achievement [51]. Such frameworks align with self-reports of flow, absorption, and joy in circumscribed interests, challenging deficit-based perspectives.

### 3.7. Psychological Development Across the Lifespan

In childhood, cognitive differences manifest as literal interpretation, preference for routines, and variable imaginative play. Adolescents face heightened EF demands and social complexity, often contributing to anxiety and camouflaging [52]. In adulthood, cognitive features may stabilize but are influenced by occupational environments and masking demands. Cognitive aging in ASD is understudied, though some evidence suggests resilience in crystallized abilities despite increased health comorbidities [53].

### 3.8. What Is Clear vs. Unclear

**Clear:** robust EF differences, variable ToM performance depending on task demands, alexithymia as a key factor in emotion processing, perceptual strengths in detail focus, and theoretical frameworks (monotropism, predictive coding) providing integrative accounts.

**Unclear:** long-term cognitive aging trajectories, generalizability of lab-based findings to everyday life, and differential contributions of co-occurring conditions to psychological profiles.

## 4. Genetic Features

### 4.1. Heritability and Genetic Architecture

ASD is among the most heritable neurodevelopmental conditions, with twin studies consistently estimating heritability between 64 and 91% [54]. Family and sibling studies confirm recurrence risks of 10–20% in siblings, highlighting strong familial aggregation [55]. However, ASD is genetically heterogeneous, with contributions from both rare, highly penetrant variants and common polygenic risk factors [56].

### 4.2. Common Variants and Polygenic Risk

Genome-wide association studies (GWASs) have identified dozens of common variants associated with autism risk, implicating synaptic function, neuronal development, and gene regulation [57,58]. Polygenic risk scores (PRSs) explain a modest proportion of variance in ASD liability but show additive effects with rare variants [59]. PRSs also overlap with other psychiatric conditions such as ADHD, schizophrenia, and depression, supporting shared neurodevelopmental pathways [60].

### 4.3. Rare Variants, CNVs, and De Novo Mutations

Rare genetic variants—including copy number variants (CNVs) and de novo single-nucleotide variants—contribute substantially to ASD, particularly in simplex families [61]. High-confidence ASD genes now exceed 200, many converging on synaptic signaling, chromatin remodeling, and transcriptional regulation [62]. Notable CNVs include 16p11.2, 22q11.2, and 15q11-q13, each conferring significant risk but with variable expressivity and pleiotropy [63]. Large-scale exome sequencing studies demonstrate that de novo loss-of-function variants in constrained genes significantly increase ASD risk [64].

### 4.4. Sex Differences and Protective Mechanisms

ASD is more frequently diagnosed in males, with ratios of ~3–4:1. Genetic findings support a “female protective effect,” whereby females require a higher mutational burden to manifest ASD traits [65]. Studies of unaffected female relatives carrying high-risk variants support this model, though biological underpinnings remain unclear [66]. Sex-differential gene expression and hormonal influences are active areas of research [67].

### 4.5. Gene–Environment Interactions

While genetics play a dominant role, gene–environment interactions contribute to ASD risk. Recent studies implicate maternal immune activation, prenatal exposures, and perinatal complications as moderators of genetic susceptibility [68,69]. For example, genetic risk may amplify the impact of maternal infection or metabolic conditions during pregnancy [70]. These interactions complicate the disentanglement of causal pathways.

### 4.6. Convergent Biological Pathways

Despite genetic heterogeneity, convergent pathways are evident. Genes implicated in ASD disproportionately affect synaptic scaffolding (SHANK3, NRXN1), chromatin remodeling (CHD8, ARID1B), and transcriptional regulation during neurodevelopment [71]. Network analyses reveal shared molecular pathways with intellectual disability and epilepsy, consistent with comorbidity patterns [72].

### 4.7. What Is Clear vs. Unclear

**Clear:** ASD has a highly polygenic and heterogeneous genetic basis; rare variants and de novo mutations explain a significant fraction of severe cases; common polygenic risk overlaps with other neuropsychiatric traits; sex differences suggest a female protective effect.

**Unclear:** the functional consequences of many risk variants, mechanisms of gene–environment interplay, and the precise biological pathways by which genetic heterogeneity produces the spectrum of behavioral and cognitive phenotypes.

## 5. Metabolic Features

### 5.1. Energy Metabolism and Mitochondrial Dysfunction

One of the most consistently reported metabolic abnormalities in ASD is mitochondrial dysfunction. Between 5 and 10% of children with ASD show biochemical evidence of mitochondrial disease, and up to 30–40% display markers of abnormal mitochondrial metabolism without meeting full diagnostic criteria [73]. Deficits in oxidative phosphorylation, increased lactate, pyruvate, and alanine levels, as well as reduced mitochondrial enzyme activities, are frequently observed [74,75]. These abnormalities are linked to impaired synaptic plasticity, disrupted neuronal energy supply, and increased vulnerability to oxidative stress [76].

### 5.2. Oxidative Stress and Redox Imbalance

Individuals with ASD often show elevated oxidative stress markers, including lipid peroxidation products, oxidized glutathione, and increased reactive oxygen species (ROS) [77]. Simultaneously, antioxidant defenses—such as glutathione peroxidase activity and reduced glutathione levels—are diminished [78]. These imbalances may contribute to neuroinflammation and disrupted signaling within cortical and subcortical regions critical for social cognition and sensory processing [79].

### 5.3. Amino Acid Metabolism and Neurotransmitter Precursors

Altered amino acid metabolism has been identified in ASD, particularly involving tryptophan, glutamate, and branched-chain amino acids [80]. Disruptions in the kynurenine pathway have been associated with excitotoxicity and altered serotonin synthesis [81]. Abnormalities in glutamine–glutamate–GABA cycling have also been reported, suggesting links between metabolism and neurotransmitter imbalances in ASD [82].

### 5.4. Folate and Methylation Cycle Abnormalities

Cerebral folate deficiency, associated with folate receptor-α autoantibodies, has been described in a subset of children with ASD [83]. Additionally, methylation cycle abnormalities—including reduced S-adenosylmethionine (SAM) and increased S-adenosylhomocysteine (SAH)—suggest impaired one-carbon metabolism and DNA methylation regulation [84]. These alterations may underlie epigenetic changes contributing to ASD phenotypes [85].

### 5.5. Lipid Metabolism and Membrane Integrity

Recent metabolomic studies highlight altered lipid metabolism in ASD, including changes in phospholipids, sphingolipids, and cholesterol [86]. These lipids are essential for myelination, synaptic vesicle formation, and membrane receptor function. Dysregulated lipid metabolism may therefore contribute to altered connectivity and signal transduction in ASD brains [87].

### 5.6. Dysautonomia in ASD

Dysautonomia, frequently reported in individuals with ASD, is classified here under metabolic dysfunction due to its strong association with mitochondrial inefficiency, oxidative stress, and impaired energy regulation. However, it is important to note that immune-mediated mechanisms (e.g., autoantibodies targeting autonomic receptors, cytokine-driven neuroinflammation) also contribute to autonomic imbalance. Thus, dysautonomia represents a cross-domain phenomenon, linking both metabolic and immune dysfunction pathways [88].

### 5.7. What Is Clear vs. Unclear

**Clear:** mitochondrial dysfunction, oxidative stress, and abnormalities in folate/methylation pathways are frequently observed in subsets of individuals with ASD. Emerging metabolomic studies identify disrupted amino acid and lipid metabolism as potential biomarkers.

**Unclear:** whether these metabolic abnormalities are causal, compensatory, or secondary to other biological processes. The heterogeneity of findings also makes it uncertain which metabolic features generalize across the spectrum, and which are limited to specific ASD subgroups.

## 6. Immunological Features

### 6.1. Neuroinflammation and Microglial Activation

Neuroinflammatory processes are among the most frequently reported immunological abnormalities in ASD. Postmortem studies reveal persistent microglial activation and increased pro-inflammatory cytokines in multiple brain regions of individuals with ASD [89]. Elevated levels of interleukin-6 (IL-6), tumor necrosis factor-α (TNF-α), and interferon-γ (IFN-γ) have been documented in both cerebrospinal fluid (CSF) and brain tissue, suggesting chronic immune dysregulation [90,91]. These findings indicate that immune system activity is tightly linked to synaptic pruning, neuroplasticity, and connectivity in ASD.

### 6.2. Peripheral Immune Abnormalities

Children with ASD frequently exhibit systemic immune dysregulation, including abnormal T-cell and B-cell responses, reduced regulatory T-cell activity, and skewed Th1/Th2 cytokine balance [92]. Increased monocyte and natural killer (NK) cell activity has also been reported, though findings remain heterogeneous [93]. Autoimmune phenomena—including elevated brain-directed autoantibodies—are described in a subset of individuals with ASD, potentially linking immune dysregulation to neurodevelopmental disruption [94].

### 6.3. Maternal Immune Activation (MIA)

Maternal immune activation during pregnancy is one of the best-established immunological risk factors for ASD. Epidemiological studies confirm that maternal infections, autoimmune conditions, and elevated inflammatory cytokines during gestation increase the risk of ASD in offspring [95]. Experimental models show that maternal IL-6 and IL-17a signaling disrupt fetal neurodevelopment, leading to behavioral phenotypes resembling ASD [96,97]. This evidence underscores the prenatal immune environment as a critical contributor to ASD pathogenesis.

### 6.4. Gastrointestinal–Immune Interactions

Gastrointestinal (GI) abnormalities are prevalent in ASD and are strongly associated with immune dysregulation. Increased intestinal permeability (“leaky gut”), altered gut microbiota composition, and mucosal immune activation have been reported [98]. These changes are linked to systemic inflammation, with microbial metabolites influencing immune signaling and brain function [99]. Dysbiosis-related immune responses are now considered part of the gut–brain–immune axis relevant to ASD symptoms.

### 6.5. Cytokine and Chemokine Profiles as Biomarkers

Several recent studies propose that cytokine and chemokine signatures could serve as biomarkers for ASD diagnosis and severity stratification. Elevated plasma levels of IL-1β, IL-6, and monocyte chemoattractant protein-1 (MCP-1) correlate with greater behavioral impairment and regression histories in children with ASD [100,101]. However, variability across studies and the lack of standardized assays limit clinical translation [88].

### 6.6. What Is Clear vs. Unclear

**Clear:** neuroinflammation, maternal immune activation, and systemic immune dysregulation are reproducibly associated with ASD. Evidence for altered cytokine profiles and autoimmune components continues to strengthen.

**Unclear:** whether immunological abnormalities are causal, secondary, or epiphenomenal. The degree to which immune modulation can be used as a therapeutic intervention remains an open question, with clinical trials of anti-inflammatory or immunomodulatory agents showing mixed results.

## 7. Speech and Language Features

### 7.1. Core Communication Challenges

Speech and language impairments are among the most recognizable features of ASD. Difficulties include delays in first words, limited phrase speech, and atypical prosody, such as unusual rhythm, pitch, or stress patterns [102]. Some individuals exhibit echolalia, immediate or delayed repetition of speech, which can serve both communicative and self-regulatory functions [103]. While not all autistic individuals have expressive language impairments, deficits in pragmatic language—the social use of communication—are nearly universal [104].

### 7.2. Structural and Functional Neural Correlates

Neuroimaging studies demonstrate atypical connectivity in brain regions underpinning language, including the superior temporal gyrus, inferior frontal gyrus (Broca’s area), and arcuate fasciculus pathways [105]. Functional MRI reveals reduced lateralization of language processing, with greater reliance on right hemisphere structures compared to neurotypical peers [106]. These patterns suggest altered specialization for speech and language, potentially contributing to both expressive and receptive challenges.

### 7.3. Early Predictors and Developmental Trajectories

Speech delay is often an early marker of ASD, but heterogeneity is high: some children acquire age-appropriate vocabulary yet struggle with pragmatics, while others present with global language impairment [107]. Early gesture use and joint attention predict later language outcomes, highlighting the importance of multimodal communication assessment [108]. Longitudinal studies show that while language skills improve in many autistic individuals, pragmatic deficits frequently persist into adolescence and adulthood [109].

### 7.4. Pragmatics and Discourse

Pragmatic impairments include challenges in conversational reciprocity, topic maintenance, and adjusting language to context [110]. Autistic speakers may demonstrate pedantic or overly formal speech, difficulty with figurative language, or reduced responsiveness to listener cues [111]. Narrative skills are also affected: autistic individuals tend to produce less coherent and less elaborated stories, with fewer causal connections [112].

### 7.5. Augmentative and Alternative Communication (AAC)

For minimally verbal individuals, augmentative and alternative communication systems—including picture exchange communication systems (PECS), speech-generating devices, and tablet-based applications—can significantly enhance expressive communication [113]. Research suggests AAC use supports both functional communication and social interaction, without impeding speech development when it emerges [114].

### 7.6. Interventions and Therapeutic Approaches

Speech–language therapy remains central to ASD intervention. Evidence-based approaches include naturalistic developmental behavioral interventions (NDBIs), milieu teaching, and pragmatic-focused therapies [115]. Parent-mediated language interventions also demonstrate effectiveness, particularly when initiated before age three [116]. Despite progress, there is ongoing debate about tailoring therapies to linguistic vs. pragmatic deficits, and how best to support minimally verbal individuals.

### 7.7. Clear vs. Unclear

**Clear:** speech and language difficulties in ASD involve both structural language (syntax, vocabulary) and pragmatic impairments, with strong neural and developmental correlates. AAC is effective for many minimally verbal children.

**Unclear:** why language development varies so widely across autistic individuals, and to what extent interventions generalize beyond structured contexts. Further research is needed to identify biomarkers predicting language trajectory and treatment responsiveness.

## 8. Genetic Findings

### 8.1. Overview

ASD is among the most heritable neurodevelopmental conditions, with twin and family studies estimating heritability between 70% and 90% [54]. Both rare, highly penetrant mutations and common polygenic variation contribute to ASD risk [117]. The current consensus is that ASD arises from a polygenic–oligogenic architecture, where combinations of risk alleles and de novo mutations interact with environmental factors to influence phenotype [118].

### 8.2. Rare Variants and De Novo Mutations

De novo mutations in genes involved in synaptic function, chromatin remodeling, and transcriptional regulation are strongly associated with ASD [119]. Key examples include CHD8, SCN2A, SYNGAP1, and ADNP, which have high penetrance but low population frequency [120]. Copy number variants (CNVs), such as deletions at 16p11.2 and 22q11.2, also confer substantial risk [121]. These rare mutations often co-occur with intellectual disability, seizures, and other neurodevelopmental disorders, highlighting pleiotropy.

### 8.3. Common Polygenic Risk

Large genome-wide association studies (GWASs) show that common variants contribute additively to ASD liability, although each variant has a small effect size [122]. Polygenic risk scores (PRSs) predict a modest proportion of variance but have revealed overlap with attention-deficit/hyperactivity disorder (ADHD), schizophrenia, and major depressive disorder, supporting the notion of shared neuropsychiatric risk architecture [123].

### 8.4. Epigenetics and Gene Regulation

Recent work emphasizes epigenetic mechanisms, such as DNA methylation, histone modifications, and noncoding RNA regulation, in shaping ASD risk [124]. Environmental exposures during pregnancy (e.g., maternal inflammation, toxins) can interact with genetic predisposition to alter epigenomic signatures in the developing brain [125]. Epigenome-wide association studies (EWASs) have identified differential methylation patterns in genes regulating neuronal differentiation and immune signaling [126].

### 8.5. Genetic Heterogeneity and Clinical Variability

Not all individuals with ASD share identifiable genetic variants, and those with the same mutation can present with vastly different phenotypes [127]. This genotypic heterogeneity reflects complex gene–environment interactions, variable expressivity, and incomplete penetrance. Clinical translation is further complicated by the overlap of autism-related genes with intellectual disability, epilepsy, and language disorders [128].

### 8.6. Translational Implications

Genetic testing, including chromosomal microarray and whole-exome sequencing, is increasingly recommended for children with ASD, especially when developmental delays or dysmorphic features are present [129]. Results can guide prognosis, comorbidity monitoring, and family planning. However, the majority of cases yield variants of uncertain significance (VUSs), limiting immediate clinical utility [130]. Future advances in integrative genomics and machine learning may improve predictive accuracy and stratification of ASD subtypes.

### 8.7. Clear vs. Unclear

**Clear:** ASD has a strong genetic basis, with both rare de novo variants and common polygenic variation contributing. Genes regulating synaptic and transcriptional processes are consistently implicated.

**Unclear:** how genetic variation translates into specific behavioral phenotypes, and the degree to which environmental and epigenetic modifiers alter penetrance. Further work is needed to connect gene-level discoveries to treatment strategies.

## 9. Metabolic Findings

### 9.1. Overview

Converging evidence indicates that mitochondrial bioenergetics, redox balance, one-carbon/folate pathways, lipid metabolism, and tryptophan–kynurenine signaling are frequently altered in ASD. Findings are heterogeneous but reproducible across independent cohorts and multi-omics studies, suggesting metabolic endophenotypes that map onto symptom severity and comorbidities [131,132,133].

### 9.2. Mitochondrial Dysfunction and Bioenergetics

Multiple studies report reduced complex I/IV activity, altered citrate-cycle flux, decreased ATP reserve capacity, and increased lactate/pyruvate ratios in blood-derived cells or brain tissue of individuals with ASD [134,135]. Single-cell and platelet/lymphocyte assays show stress-induced mitochondrial hyper- or hyporesponsivity, consistent with a subset having primary mitochondrial dysfunction and others showing secondary, context-dependent changes [134]. Clinically, bioenergetic abnormalities correlate with motor delay, fatigue, GI symptoms, and seizure susceptibility [135].

### 9.3. Oxidative Stress and Redox Balance

Meta-analyses report increased lipid peroxidation products (e.g., MDA, F2-isoprostanes), reduced glutathione (GSH), and lower GSH:GSSG ratios, with effect sizes largest in cohorts with higher autistic trait burden [135]. Redox imbalance interacts with mitochondrial inefficiency, forming a self-reinforcing loop that can impair synaptic plasticity and interneuron physiology [82,136].

### 9.4. One-Carbon Metabolism and Folate Pathways

Disturbances in folate transport and methylation capacity—particularly folate receptor-α autoantibodies and low CSF 5-MTHF in a subset—remain robust findings and relate to language delay and irritability [137]. Trials and series suggest that folinic acid (leucovorin) can benefit language and adaptive functioning in biomarker-positive children, though effect sizes vary and replication with stratification is needed [137,138].

### 9.5. Tryptophan–Kynurenine and Neuroimmune Crosstalk

Several cohorts show lower circulating tryptophan, increased kynurenine–tryptophan ratios, and shifts toward neuroactive metabolites (e.g., quinolinic/kynurenic acid), consistent with indoleamine 2,3-dioxygenase (IDO) activation under inflammatory tone [139]. These profiles associate with sensory abnormalities, sleep disturbance, and anxiety features [139,140].

### 9.6. Lipidomics and Membrane Composition

Lipidomic studies indicate altered phosphatidylcholine/phosphatidylethanolamine balance, decreased long-chain polyunsaturated fatty acids, and perturbed sphingolipid metabolism, which relate to executive dysfunction and social affect scores [141]. Membrane lipid remodeling may modulate receptor trafficking and excitatory–inhibitory balance.

### 9.7. Carnitine and Acyl-Carnitine Signatures

Abnormal acyl-carnitine profiles (short- and long-chain species) and lower free carnitine have been reported, supporting impaired fatty acid transport into mitochondria in a subset [142]. Pilot supplementation studies suggest modest gains in lethargy and social withdrawal, with best responses in children with baseline carnitine deficits [142].

### 9.8. Microbiome–Metabolome Axis

Integrated microbiome–metabolome analyses (fecal SCFAs, bile acids, aromatic amino acid catabolites) reveal distinct clusters in ASD that associate with GI symptoms and adaptive behavior [143]. Short-chain fatty acids (e.g., propionate) and p-cresol derivatives have been implicated in microglial priming and synaptic pruning pathways; however, inter-study variability underscores the need for standardized diets and sampling [143,144].

### 9.9. Metabolomics for Stratification and Biomarkers

Untargeted and targeted metabolomics with machine learning classifiers achieve AUCs ~0.75–0.90 for case–control discrimination in held-out sets, but cross-platform reproducibility and external validation remain barriers to clinical deployment [132,145]. Best practices emphasize multi-site replication, pre-registration, and harmonized pipelines.

### 9.10. Therapeutic Implications

- **Folinic acid**: language/communication gains in FRα-autoantibody-positive children; confirmatory, biomarker-stratified RCTs encouraged [137,138].
- **L-carnitine**: possible improvements in fatigue/behavior for low baseline carnitine or abnormal acyl-carnitines [142].
- **Coenzyme Q10, NAC, omega-3s**: mixed but generally favorable safety; signal for redox and irritability endpoints in selected subgroups [82,141].
- **Dietary and microbiome interventions**: emerging benefits in GI subtypes; prioritize rigorous, blinded, diet-controlled trials with metabolomic endpoints [143,144].

### 9.11. What Is Clear vs. Uncertain

**Clear:** metabolic heterogeneity is real; reproducible abnormalities occur across bioenergetics, redox, folate, lipid, and kynurenine pathways; metabolomics can aid stratification.

**Uncertain:** which metabolic signatures are causal vs. compensatory; durability and generalizability of metabolic interventions; best biomarker panels for clinical decision support.

## 10. Immunological Findings

### 10.1. Overview

Immune dysregulation has emerged as one of the most consistent biological signatures in ASD. Altered cytokine profiles, neuroimmune crosstalk, and evidence of maternal immune activation contribute to the view of ASD as a condition with a strong immunological endophenotype [146,147,148].

### 10.2. Peripheral Immune Alterations

Meta-analyses and large cohort studies demonstrate increased circulating pro-inflammatory cytokines (IL-6, TNF-α, IL-1β) and altered chemokine signatures (e.g., CCL2, CXCL8). These profiles associate with sleep disturbance, aggression, and repetitive behaviors [149,150]. Regulatory T-cell (Treg) imbalance and altered NK-cell activity are also described, suggesting compromised immune regulation [151].

### 10.3. Neuroimmune Interactions and Microglia

Postmortem and in vivo imaging studies confirm microglial priming and activation in ASD brains. Transcriptomic data indicate persistent M1-like pro-inflammatory signatures, while reduced homeostatic microglial programs impair synaptic pruning and plasticity [152,153]. PET imaging with TSPO ligands reveals region-specific microglial activation linked to social cognition deficits [152].

### 10.4. Maternal Immune Priming

While epidemiological and animal data remain promising, prenatal infection, autoimmune disease, or elevated maternal cytokines (especially IL-6 and IL-17a) increase offspring ASD risk [154,155,156]. Human placental studies report elevated inflammatory markers and altered trophoblast gene expression, supporting intrauterine immune priming as a mechanism [155].

### 10.5. Autoimmunity and Brain-Directed Antibodies

Maternal antibodies targeting fetal brain antigens (MAR autism antibodies) are confirmed in ~10% of ASD cases, linked to language delay and macrocephaly [156]. New evidence shows anti-synaptic and anti-folate receptor antibodies correlating with irritability and regression subtypes [157].

### 10.6. Gut–Immune Axis

Children with ASD frequently show mucosal immune activation, altered IgA/IgG responses to commensals, and increased intestinal permeability markers [158]. These correlate with GI symptoms and irritability, implicating the microbiome–immune crosstalk in phenotype expression [159].

### 10.7. Neuroinflammation Biomarkers

CSF and blood biomarkers—soluble TREM2, neopterin, complement proteins—emerge as promising but require validation. Cytokine/chemokine ratios may serve as state vs. trait markers, but reproducibility across platforms is limited [160,161].

### 10.8. Immunomodulatory Interventions

- **IVIG**: some open-label studies suggest gains in socialization/communication; RCTs underpowered and heterogeneous [162].
- **Anti-inflammatory agents** (minocycline, sulforaphane, resolvins): modest but reproducible behavioral signal in small trials [163,164].
- **Targeted biologics** (anti-IL-6, anti-IL-17): under preclinical testing; no human RCTs yet [154].
- **Microbiota-directed interventions**: reported to reduce both GI and behavioral symptoms, but placebo-controlled reproducibility is limited [159].

### 10.9. What Is Clear vs. Uncertain

**Clear:** peripheral and central immune dysregulation is reproducible; maternal immune activation increases risk; immune phenotypes track with symptom dimensions.

**Uncertain:** which immune profiles are causal vs. compensatory; which patients benefit most from immunomodulation; reproducibility of immune biomarkers for clinical stratification.

## 11. Neurological and Brain Connectivity Findings

### 11.1. Structural White Matter Alterations (DTI/Structural MRI)

Diffusion studies continue to show robust, region-specific microstructural differences in ASD across long-range association tracts (e.g., corpus callosum, SLF/ILF, cingulum) with age-dependent trajectories. In preschool-aged children, automated fiber quantification (AFQ) analyses demonstrate early alterations in multiple tracts, supporting the view that atypical connectivity is already present before formal schooling and may contribute to cascading developmental effects [165].

### 11.2. Functional Connectivity (Resting/Task fMRI, MEG/EEG)

Findings remain heterogeneous, with both hypo- and hyperconnectivity reported across the default mode, salience, and frontoparietal systems, varying by age, cognitive state, and symptom profile. Emerging work underscores context dependence (rest vs. task), heterogeneity of subtypes, and developmental nonlinearity.

### 11.3. Network Topology (Graph Theory)

Graph theory is a mathematical framework used to study complex networks, including the brain’s structural and functional connections. In this framework, the brain is modeled as a set of nodes (brain regions) connected by edges (structural pathways or functional correlations). Several key properties are commonly reported in autism studies:

#### 11.3.1. Small-Worldness

A balance between local clustering (specialized processing within regions) and global efficiency (integration across regions). Healthy brains typically show “small-world” organization, which supports both specialized and distributed processing.

#### 11.3.2. Global Efficiency

Reflects how efficiently information is exchanged across the whole network. Reduced global efficiency in ASD suggests less effective integration of information between distant brain regions.

#### 11.3.3. Hubness

Certain nodes act as hubs—highly connected regions that coordinate information flow. In ASD, hubs often shift from association cortices (frontal, parietal) to more localized or sensory regions, indicating altered communication strategies.

#### 11.3.4. Integration vs. Segregation

Typical brain networks maintain a dynamic balance between integrated processing across regions and segregated, specialized subsystems. Studies in ASD often report reduced integration and increased segregation, potentially underlying difficulties in combining information across cognitive domains.

Developmental studies further show that these graph metrics follow atypical trajectories in ASD, with some measures (e.g., efficiency) lagging behind neurotypical maturation. This suggests that differences in network topology may reflect a developmental divergence rather than a static deficit. Together, these findings highlight how graph theory provides a quantitative lens to describe large-scale brain organization in ASD and its impact on information processing and cognition.

### 11.4. Sensory and Salience Network Contributions

Altered coupling among sensory cortices, the insula, and temporoparietal regions may relate to sensory hyper/hyporeactivity and difficulties with salience attribution. This aligns with behavioral phenotypes (e.g., sensory seeking/avoidance) and could mediate downstream social cognitive effects.

### 11.5. Task-Dependent Reconfiguration and Idiosyncrasy

Task engagement often yields atypical state reconfiguration and higher inter-individual idiosyncrasy of connectivity in ASD compared with controls, particularly in social cognitive tasks. Such idiosyncrasy may obscure group-level averages and argue for person-specific analytic approaches.

### 11.6. Underconnectivity Perspective

A broad “underconnectivity” account—reduced coordination among distant cortical regions supporting higher cognition—continues to have support from convergent fMRI evidence, especially in association networks subserving language, executive, and social functions [166]. At the same time, local hyperconnectivity and state-dependent hypersynchrony have been documented, suggesting a mixed model with circuit- and state-specific effects rather than a single global pattern.

Taken together, ASD shows (i) early-appearing white matter differences (including in preschool children via AFQ DTI [165]), (ii) mixed hypo/hyper-functional connectivity that depends on development and task state, and (iii) network-level alterations in efficiency and hub organization. These converge on a model of atypical integration/segregation dynamics across development, with implications for individualized intervention timing and targets.

### 11.7. What Is Clear vs. Uncertain

**Clear**: consistent evidence of altered white matter integrity and atypical connectivity in ASD, including in early childhood.

Disruption of long-range connectivity with simultaneous increases in short-range connections.

**Mixed/Context-dependent**: patterns of hyper- versus hypoconnectivity vary depending on age, task, and region examined.

Functional implications of oscillatory abnormalities are not yet fully established.

**Emerging**: use of connectivity-based biomarkers for diagnosis and prognosis. Integration of multimodal imaging (DTI, fMRI, EEG/MEG) to provide a systems-level view of neural circuitry.

## 12. Developmental and Maturational Factors

ASD arises from the interplay of genetic, neurobiological, and environmental factors within the context of developmental and maturational processes across the lifespan. These influences shape brain growth, hemispheric specialization, hormonal transitions, and social adaptation, highlighting ASD as a dynamic condition with evolving manifestations rather than a static phenotype.

### 12.1. Early Brain Growth and Critical Periods

Abnormal early brain growth remains one of the most replicated findings in ASD. Infants later diagnosed with ASD often demonstrate accelerated head circumference and brain volume expansion during the first two years of life, followed by atypical plateauing or deceleration [34,166]. Neuroimaging reveals early overgrowth in frontal and temporal cortices and amygdala volume, potentially contributing to language and social impairments [167]. These findings point to disrupted critical periods of synaptic pruning, dendritic arborization, and myelination [168,169].

### 12.2. Hemisphericity and Developmental Asymmetries

Typical brain development involves functional and structural asymmetries between hemispheres, particularly left-hemisphere dominance for language and right-hemisphere specialization for visuospatial and social-emotional processing. In ASD, multiple studies have reported reduced or atypical hemispheric asymmetry, including diminished leftward lateralization for language, aberrant right-hemisphere dominance in auditory processing, and altered interhemispheric connectivity via the corpus callosum [170,171]. Such deviations may underlie speech–language difficulties, prosody impairments, and atypical social-emotional cue processing. Longitudinal work suggests these asymmetries emerge early and persist, implicating prenatal and early postnatal neurodevelopmental mechanisms [172].

### 12.3. In Utero Influences

Gestational factors play a critical role in shaping ASD risk. Maternal immune activation (MIA) has been linked to altered fetal neurodevelopment via pro-inflammatory cytokines crossing the placenta [173]. Maternal metabolic conditions such as obesity, diabetes, and dyslipidemia increase the likelihood of ASD, possibly through impacts on placental nutrient transport and oxidative stress [174]. Endocrine exposures—including maternal thyroid hormone dysregulation and elevated cortisol from prenatal stress—also alter neurodevelopmental trajectories [175]. These in utero influences may help explain early neuroanatomical changes, hemispheric asymmetry, and differences in excitatory/inhibitory balance observed in ASD brains.

### 12.4. Postpartum Effects

Postnatal development is further shaped by environmental exposures and caregiving. Factors such as early sensory environment, breastfeeding and nutrition, caregiver responsiveness, and early-life stress modulate neuroplasticity and socio-communicative development [176]. Infants with ASD often demonstrate atypical gaze, joint attention, and sensory reactivity in the first year of life, which may interact with caregiver responses and shape ongoing social-cognitive development [177]. Epigenetic mechanisms, including DNA methylation influenced by caregiving quality, have been identified as mediators of early risk and resilience [178].

### 12.5. Cognitive and Behavioral Development

Developmental trajectories vary widely. Some children show developmental arrest or plateauing in skills, whereas others experience regression with loss of previously acquired language or social behaviors [179]. Motor delays often precede or co-occur with communication deficits [180]. While certain cognitive strengths (e.g., visuospatial processing) remain stable, executive function, social cognition, and adaptive behaviors often diverge further from neurotypical peers over time [181,182].

### 12.6. Puberty and Hormonal Influences

Adolescence introduces profound neuroendocrine changes. Pubertal surges in sex steroids can exacerbate ASD symptoms, including emotional dysregulation, anxiety, and sensory sensitivity [183]. Neuroimaging shows altered synaptic pruning and reorganization during adolescence, particularly in prefrontal–limbic circuitry [184]. Sex-specific patterns emerge: autistic females may experience delayed or atypical onset of social difficulties and distinct hormonal profiles [185].

### 12.7. Lifespan Development and Aging

Although most research emphasizes childhood, ASD persists into adulthood and interacts with aging processes. Evidence suggests increased vulnerability to accelerated cognitive aging and neurodegenerative conditions, including Parkinson’s disease and Alzheimer’s pathology, in some individuals with ASD [186,187]. However, domains such as sensory processing may remain preserved, suggesting complex trajectories of vulnerability and resilience [188].

### 12.8. Developmental Plasticity and Intervention Windows

Sensitive periods for intervention exist throughout development. Early behavioral and language interventions can yield enduring effects [189]. More recent evidence indicates that adolescence and adulthood also retain substantial plasticity, with measurable cognitive and neural gains following targeted therapies [190,191]. Recognizing developmental stage-specific plasticity enables tailoring of interventions to maximize outcomes across the lifespan.

### 12.9. What Is Clear vs. Uncertain

**Clear:** early brain overgrowth and atypical deceleration are consistent. Hemispheric asymmetry reductions are well replicated, particularly in language networks. Maternal immune activation and metabolic conditions are established in utero risk factors. Puberty and hormonal transitions represent sensitive periods for symptom fluctuation.

**Mixed/Context-dependent:** evidence for accelerated cognitive aging in ASD remains inconsistent. Postpartum influences (nutrition, caregiver interaction) vary across contexts and are difficult to disentangle. Hemispheric asymmetry findings differ depending on task modality and developmental stage.

**Emerging:** epigenetic mechanisms linking prenatal and postnatal environments to neurodevelopment are being actively investigated. Studies of neurodegeneration in autistic older adults remain limited but growing.

Integration of hemispheric connectivity and in utero exposures offers a promising avenue for clarifying developmental origins.

## 13. Discussion

Autism spectrum disorder (ASD) represents one of the most complex and heterogeneous neurodevelopmental conditions, spanning behavioral, cognitive, genetic, metabolic, immunological, and neurobiological dimensions. Across these domains, several findings are now well established, whereas others remain equivocal, reflecting both methodological variability and the inherent diversity of autistic presentations.

From a behavioral standpoint, the literature is robust in documenting that social communication differences and restricted, repetitive behaviors are defining features that persist across the lifespan. These traits, however, are not uniform but instead reveal considerable variability linked to sex, language level, co-occurring conditions, and developmental stage [1,2]. Evidence for camouflaging behaviors, particularly among females and gender-diverse individuals, has grown substantially, highlighting both adaptive strategies and the cost of such masking in terms of mental health outcomes [12,13,18]. Nevertheless, uncertainties remain regarding the long-term impact of camouflaging and its relationship to burnout and quality of life, as well as the trajectories of autistic traits in later adulthood, which are only beginning to be studied systematically [15,16,17].

In the psychological domain, consistent evidence points to executive function differences, especially in flexibility, planning, and working memory, which have downstream consequences for adaptive functioning [11,11,40]. At the same time, theories such as weak central coherence, theory of mind differences, and predictive coding frameworks emphasize variability rather than universal deficits [4,38,49]. Emotional processing and regulation also emerge as significant areas of difference, yet findings are complicated by the role of alexithymia, sensory sensitivities, and co-occurring anxiety [42,43,44,45]. While there is clear support for perceptual strengths and cognitive styles such as monotropism and systemizing [50,51], the extent to which these confer resilience versus vulnerability remains an open question.

Genetic studies have unequivocally established ASD as highly heritable, with contributions from both common polygenic risk factors and rare de novo variants [54,55,56,57,118,119,120,121]. Well-replicated findings implicate pathways in synaptic scaffolding, chromatin remodeling, and transcriptional regulation [62,71]. Nonetheless, translating this genomic architecture into phenotypic expression has proven difficult, as identical mutations may yield diverse outcomes depending on background variation and environmental influences [126,127]. Similarly, the female protective effect is supported by increasing evidence, but its biological mechanisms remain unclear [65,66].

Metabolic and immunological studies have provided some of the most provocative but also heterogeneous findings. Mitochondrial dysfunction, oxidative stress, and abnormalities in folate and methylation cycles are consistently reported in subsets of individuals with ASD [73,77,83]. Parallel evidence indicates immune dysregulation, neuroinflammation, and maternal immune activation as recurrent themes across both human and animal studies [88,89,90,94,95,96]. Yet, whether these features represent primary causal mechanisms or secondary consequences of other processes is unresolved, and replication across independent cohorts remains a challenge [79,92,99]. Moreover, while interventions targeting metabolic or immune pathways—such as folinic acid or immunomodulation—show early promise, reproducibility and biomarker stratification remain barriers to clinical translation [136,161,162,163].

Language and communication differences continue to be central in ASD, with pragmatic impairments highlighted as nearly universal, even in individuals with strong structural language [101,109]. Neuroimaging evidence consistently shows atypical lateralization and altered connectivity within language networks [104,105]. Augmentative and alternative communication has proven effective for minimally verbal individuals, representing one of the clearest examples of evidence-based intervention translating into improved participation and quality of life [112,113]. Still, the wide heterogeneity in language development trajectories, ranging from early fluency to profound delays, underscores gaps in identifying reliable predictors of outcome [106,108].

Neuroimaging and network neuroscience have expanded the understanding of ASD as a condition of altered connectivity. Diffusion tensor imaging and functional MRI studies converge on early structural and functional atypicalities, with evidence for both hypo- and hyperconnectivity depending on region, task state, and developmental stage [164,192]. Graph theory approaches provide a quantitative lens, revealing differences in network efficiency, hub distribution, and integration–segregation balance. However, such metrics require cautious interpretation given variability across analytic pipelines and the challenges of linking network-level differences directly to clinical symptoms. The integration of multimodal imaging and electrophysiology may ultimately clarify these associations.

Finally, developmental perspectives emphasize that ASD must be understood as a dynamic condition unfolding across sensitive periods. Early brain overgrowth and atypical hemispheric specialization are replicated findings, as are the impacts of maternal immune activation and metabolic conditions during gestation [169,172,173,192]. Yet postpartum influences such as caregiver responsiveness, sensory environment, and epigenetic mechanisms remain less consistently characterized [175,176,177]. Adolescence introduces new complexities, including the influence of hormonal transitions, social demands, and risks of mental health comorbidities [182,183,184]. Lifespan research remains particularly sparse, with questions about cognitive aging and vulnerability to neurodegenerative processes largely unanswered [185,186]. Table 1 summarizes our current knowledge base to clearly indicate where we are in our understanding of ASD and where we have yet to go.

Taken together, what is clear is that ASD arises from a convergence of genetic, neurobiological, metabolic, and environmental influences, interacting dynamically with developmental trajectories. What remains equivocal is the relative weighting of these influences across individuals, the causal sequencing of observed biological abnormalities, and the extent to which interventions targeting specific pathways yield durable, generalizable benefits. Future research must prioritize longitudinal designs, multi-omics integration, and ecological assessment methods to bridge the gap between laboratory findings and lived experience.

## 14. Conclusions

The accumulated evidence underscores both the progress and persistent challenges in understanding autism spectrum disorder (ASD). Findings across neurodevelopmental, cognitive, and behavioral domains converge to demonstrate that ASD is not a singular entity but a heterogeneous condition characterized by variability in presentation, developmental course, and underlying mechanisms [1,12,17]. Established results from large-scale and longitudinal investigations have clarified that atypicalities in executive functioning, social cognition, and sensory processing are core features that manifest early and often persist into adolescence and adulthood [3,6,10]. At the same time, neuroimaging and electrophysiological research have identified consistent alterations in connectivity and cortical dynamics, supporting the view of ASD as a disorder of distributed neural networks rather than isolated regional deficits [7,14].

Yet, despite such advances, major gaps remain. The translation of neurobiological markers into clinically actionable diagnostics or interventions is limited, and findings from small-scale or preliminary studies often lack replication across diverse samples [4,9,15]. Moreover, heterogeneity within ASD continues to obscure the identification of universal mechanisms, highlighting the need for refined subtyping approaches and multimodal integration of behavioral and biological measures [2,8,13]. Equivocal results persist, particularly in areas such as the role of compensatory mechanisms, the longitudinal stability of cognitive trajectories, and the generalizability of emerging computational models of cognition [5,11,16].

Overall, the literature reflects a field at a pivotal juncture. Robust and replicated findings have provided a foundation for theoretical models of atypical neurodevelopment, yet the variability of outcomes underscores the importance of caution in drawing broad generalizations. Future progress will depend on integrating large-scale data resources, pre-registered hypotheses, and multimodal methodologies that bridge cognitive neuroscience with clinical practice. In doing so, research can move closer to delineating not only what is atypical in ASD but also what is adaptive, thereby advancing both scientific understanding and meaningful clinical outcomes for autistic individuals and their families.

## Figures and Tables

**Table 1:** Knowledge status across ASD domains.

Domain	What Is Known	What Is Unknown	What Is Equivocal
**Behavioral**	Social communication differences and restricted/repetitive behaviors are core features; heterogeneity by sex, language, and co-occurring conditions is established; camouflaging is common, especially in females; autistic burnout is recognized.	Long-term effects of camouflaging and burnout on mental health and quality of life; trajectories of autistic traits in older adulthood.	Mapping behavioral subtypes to biology; ecological validity of new digital/eye-tracking metrics; impact of camouflaging on adaptive outcomes.
**Psychological**	Executive function differences (flexibility, working memory, inhibition) are robust; alexithymia explains much emotion recognition variance; perceptual strengths in detail focus; frameworks like monotropism and predictive coding provide explanatory models.	Aging trajectories in cognition; generalization of lab-based findings to daily life; contribution of co-occurring conditions to profiles.	Extent to which perceptual/cognitive styles confer resilience vs. vulnerability; variability of theory of mind across contexts.
**Genetic**	High heritability (64–91%); >200 ASD risk genes; both common polygenic and rare de novo variants implicated; female protective effect supported.	Functional consequences of many variants; mechanisms of gene–environment interplay; how genetic heterogeneity maps to phenotype.	Extent of pleiotropy with ID/epilepsy; clinical utility of polygenic risk scores; biological basis of sex differences.
**Metabolic**	Mitochondrial dysfunction, oxidative stress, and folate/methylation abnormalities in subsets; altered amino acid and lipid metabolism reproducibly reported.	Whether metabolic abnormalities are causal, compensatory, or secondary; which signatures generalize across the spectrum.	Efficacy of metabolic interventions (folinic acid, carnitine, omega-3s) across ASD subtypes; reproducibility of biomarker-based stratification.
**Immunological**	Neuroinflammation, maternal immune activation, and systemic immune dysregulation reproducibly linked to ASD; altered cytokine/chemokine profiles identified.	Causality of immune abnormalities; optimal targets for immunomodulation; which patients benefit from immune-directed interventions.	Mixed results of immunomodulatory trials (e.g., IVIG, sulforaphane); reproducibility of immune biomarkers across labs.
**Language and Communication**	Pragmatic deficits nearly universal; atypical lateralization and altered connectivity in language networks; AAC shown effective for minimally verbal children.	Predictors of language development trajectories; biomarkers for treatment responsiveness.	Extent to which interventions generalize beyond structured settings; variability in outcomes of speech–language therapies.
**Neurological and Connectivity**	Altered white matter integrity and atypical functional connectivity consistently reported; atypical network efficiency and hub distribution via graph theory.	Functional meaning of oscillatory abnormalities; developmental progression of connectivity changes.	Patterns of hypo- vs. hyperconnectivity vary by age, task, and region; person-specific variability obscures group-level effects.
**Developmental and Maturational**	Early brain overgrowth; atypical hemispheric asymmetry; maternal immune activation and metabolic risk factors robust; puberty as a sensitive period.	Postpartum influences (nutrition, caregiving) and their epigenetic mediation; cognitive aging and risk of neurodegeneration.	Evidence for accelerated cognitive aging is mixed; hemispheric asymmetry findings vary by developmental stage and modality.

## Data Availability

No data were collected or employed in this study.

## Abbreviations

The following abbreviations are used in this manuscript:

AAC	Augmentative and alternative communication
ADHD	Attention-deficit/hyperactivity disorder
ADI-R	Autism Diagnostic Interview-Revised
ADOS	Autism Diagnostic Observation Schedule
AFQ	Automated fiber quantification
ASD	Autism spectrum disorder
CNV	Copy number variants
CSF	Cerebrospinal fluid
DTI	Diffusion tensor imaging
EEG	Electroencephalogram
EWASs	Epigenome-wide association studies
fMRI	Functional magnetic resonance imaging
GI	Gastrointestinal
GSH	Glutathione
GWASs	Genome-wide association studies
ID	Intellectual disability
IDO	Indoleamine 2,3 dioxygenase
IVIG	Intravenous immunoglobulin
MAR	Maternal antibody-related
MCP-1	Monocyte chemoattractant protein-1
MEG	Magnetoencephalography
MIA	Maternal immune activation
MRI	Magnetic resonance imaging
NDBI	Naturalistic behavioral intervention
NK	Natural killer cells
PECS	Picture exchange communication system
PRSs	Polygenic risk scores
ROS	Reactive oxygen species
RRB	Restrained repetitive behaviors
SAH	S-adenosylhomocysteine
SAM	S-adenosylmethionine
ToM	Theory of Mind
Treg	Regulatory T cells
TNF	Tumor necrosis factor-α
VUSs	Variants of uncertain significance
